# Functional assessment of somatic *STK11* variants identified in primary human non-small cell lung cancers

**DOI:** 10.1093/carcin/bgab104

**Published:** 2021-11-19

**Authors:** Liam L Donnelly, Tyler C Hogan, Sean M Lenahan, Gopika Nandagopal, Jenna G Eaton, Meagan A Lebeau, Cai L McCann, Hailey M Sarausky, Kenneth J Hampel, Jordan D Armstrong, Margaret P Cameron, Nikoletta Sidiropoulos, Paula Deming, David J Seward

**Affiliations:** 1 Department of Pathology and Laboratory Medicine, University of Vermont College of Medicine, Burlington, VT, USA; 2 Department of Biomedical and Health Sciences, University of Vermont College of Nursing and Health Sciences, Burlington, VT, USA; 3 University of Vermont, Burlington, VT, USA; 4 University of Vermont Cancer Center, Burlington, VT, USA

## Abstract

Serine/Threonine Kinase 11 (STK11) encodes an important tumor suppressor that is frequently mutated in lung adenocarcinoma. Clinical studies have shown that mutations in STK11 resulting in loss of function correlate with resistance to anti-PD-1 monoclonal antibody therapy in KRAS-driven non-small cell lung cancer (NSCLC), but the molecular mechanisms responsible remain unclear. Despite this uncertainty, STK11 functional status is emerging as a reliable biomarker for predicting non-response to anti-PD-1 therapy in NSCLC patients. The clinical utility of this biomarker ultimately depends upon accurate classification of STK11 variants. For nonsense variants occurring early in the STK11 coding region, this assessment is straightforward. However, rigorously demonstrating the functional impact of missense variants remains an unmet challenge. Here we present data characterizing four STK11 splice-site variants by analyzing tumor mRNA, and 28 STK11 missense variants using an in vitro kinase assay combined with a cell-based p53-dependent luciferase reporter assay. The variants we report were identified in primary human NSCLC biopsies in collaboration with the University of Vermont Genomic Medicine group. Additionally, we compare our experimental results with data from 22 *in silico* predictive algorithms. Our work highlights the power, utility and necessity of functional variant assessment and will aid STK11 variant curation, provide a platform to assess novel STK11 variants and help guide anti-PD-1 therapy utilization in KRAS-driven NSCLCs.

## Teaser

Functional assessment of under- and undescribed clinical *STK11* variants highlights the limitations of current *in silico* predictive algorithms. Successful implementation of personalized genomic medicine will rely on rapid and accurate variant classification.

## Introduction

Each year ~230 000 people in the USA are diagnosed with lung cancer, 85% of which will be histologically classified as non-small cell lung cancer (NSCLC) (1). Despite dedicated work focused on improving treatment and care, the overall prognosis for patients with lung cancer remains poor. Tumors harboring druggable oncogenic mutations have demonstrated improved initial responses with use of targeted therapies, but due to the acquisition of therapy resistance, the overall 5-year survival rate for lung cancer patients remains ~15–20% (2,3). Current Food and Drug Administration-approved immune oncologic approaches target mechanisms known to manifest in tumor immune-evasion. Pembrolizumab and nivolumab are immune checkpoint inhibitors and represent examples of monoclonal antibody therapy designed to disrupt the PD-L1/PD-1 interaction that governs one common tumor immune-evasion strategy (4–6). Unfortunately, tools that reliably predict which patients will benefit from immune checkpoint inhibitor therapy do not yet exist. Approximately 30% of NSCLC patients respond to anti-PD-1 therapy, but we cannot confidently predict who those patients will be prior to treatment (7,8). This knowledge gap is a critical hurdle limiting effective use of current anti-PD-1 therapies and impeding development of next-generation interventions. These observations underscore a demonstrable need to develop biomarkers able to discriminate which NSCLC patients will respond to immuno-therapies while simultaneously highlighting the urgancy for novel therapeutic approaches addressing the 70% non-responder rate.

Recently, the functional status of Serine/Threonine Kinase 11 (*STK11*) has emerged as a putative prognostic and therapeutic biomarker in patients diagnosed with KRAS-driven NSCLC (9,10). Specifically, clinical studies have demonstrated that nonsense variants in *STK11*, which predict loss of STK11 function, correlate with primary resistance to PD-1 axis inhibitors in *KRAS*-mutant lung adenocarcinoma patients (10,11). This combined genotype occurs in ~15% of NSCLCs and is associated with a worse prognosis and increased rates of metastasis (9,10,12,13). *STK11*, formerly called Liver Kinase B1 (*LKB1*), encodes an important tumor suppressor that regulates numerous intracellular signaling networks impacting metabolism, proliferation and cell morphology (14). Heterozygous germline *STK11* LoF variants are pathognomonic for Peutz-Jeghers syndrome, an autosomal dominant heritable cancer predisposition syndrome characterized by melanocytic macules of the oral mucosa and gastrointestinal hamartomas (15). While somatic mutation of *STK11* is rare in most cancers, it represents one of the most frequent genomic alterations in NSCLC (16,17). Regarding its molecular function as a serine/threonine kinase, STK11 operates in a heterotrimeric complex with the pseudo-kinase STRADα and the scaffolding protein MO25 (18,19). Its direct characterized phosphorylation targets include 13 members of the microtubule affinity-regulating kinases family, AMPK being the most thoroughly studied (20,21). Additional downstream effectors include the p53 signaling axis. STK11 has been shown to physically associate with p53 in the nucleus and activate p53-mediated transcriptional activity to regulate proliferation and apoptosis (22,23). The mechanism(s) governing STK11-dependent activation of p53 remain unclear and may occur directly via STK11-mediated phosphorylation of p53 on Ser15 and Ser392 (23–25), or indirectly through the activation of AMPK and NUAK1 (26). In either case, p53 activation relies on intact STK11 kinase function. The absence or impairment of STK11 results in reduced p53-dependent transcriptional activation (27,28).

The widespread implementation of next-generation sequencing assays to evaluate tumor mutations for the purpose of targeted therapy utilization has resulted in a significant increase in the number of variants identified in critical cancer genes. In particular, rare variants identified outside established ‘hot-spots’ are a common finding. When novel or rare variants are noted, there is frequently little published information regarding the functional consequence, and these alterations are classified as variants of uncertain significance (VUS). The clinical application of variant analysis relies on the ability to assign a functional consequence to each identified variant. For tumor suppressor genes like *STK11*, interpreting nonsense variants occurring early in the coding region is trivial. However, predicting the functional impact of other variant classes remains a challenge. *STK11* variants that occur at conserved non-coding splice-sites are often assumed to disrupt splicing and generate non-productive mRNA molecules, effectively recapitulating STK11 loss of function (LoF). However, this assumption is rarely backed by rigorous experimental evidence. To confidently determine the functional consequence of splice-site variants the mRNA must be sequenced. Taking this approach reveals the molecular impact of the splice-site variant, something that cannot be done reliably based on DNA sequence alone (29–32). The functional assessment of missense variants poses a separate and more complex set of challenges. Traditionally, *in vitro* assays with recombinant proteins have been used to assess variant impact. This assumes the activity of the protein in question is understood well enough to assess function, and that its behavior *in vitro* recapitulates that *in vivo*. While there are inherent limitations to this approach, it does provide a direct avenue to identify variants that alter protein function.

Here we present data characterizing the functional status of four *STK11* splice-site variants and 28 *STK11* missense variants (Table 1) detected in primary human KRAS-driven NSCLC biopsy specimens. Given the clinical data demonstrating patients with KRAS-driven NSCLC lacking functional STK11 respond poorly to anti-PD-1 mono-therapy (10), our analyses of un- and under-characterized *STK11* variants provides critical pre-clinical evidence that can be used to help guide oncologic care for the ~15 000 patients diagnosed annually with KRAS-driven, *STK11*-mutated NSCLC. In addition, by contrasting our results with predictions made by 22 *in silico* algorithms we demonstrate the need to develop and improve tools capable of rapidly assessing the functional impact of novel gene variants to achieve the goal of personalized genomic medicine.

**Table 1.** Genomic locations for the STK11 variants evaluated

STK11 splice-site variant ID	c.SYNTAX	g.SYNTAX	Intron location; donor/acceptor
V1	NM_000455.4:c.598-1G>A	chr19:g.1220579G>A	4; splice acceptor
V2	NM_000455.4:c.464+1G>T	chr19:g.1219413G>T	3; splice donor
V3	NM_000455.4:c.862+1G>A	chr19:g.1221340G>A	6; splice donor
V4	NM_000455.4:c.465-2A>C	chr19:g.1220370A>C	3; splice acceptor
STK11 Missense Variant	p.SYNTAX	g.SYNTAX	ClinVar accession #
S31F	NP_000446.1:p.S31F	chr19:g.1207004C>T	—
G56W	NP_000446.1:p.G56W	chr19:g.1207078G>T	—
Y60*	NP_000446.1:p.Y60*	chr19:g.1207092C>G	VCV000428749
K84del	NP_000446.1:p.K84_K84del	chr19:g.1207153_1207155delAAG	VCV000141849
R104G	NP_000446.1:p.R104G	chr19:g.1218435A>G	VCV000182905
Q112E	NP_000446.1:p.Q112E	chr19:g.1218459C>G	VCV000185602
F148S	NP_000446.1:p.F148S	chr19:g.1219391T>C	—
G155R	NP_000446.1:p.G155R	chr19:g.1219411G>A	VCV000234791
G156_S169del	NP_000446.1:p.G156_S169_del	chr19:g.1220370A>C	VCV000428767
G163R	NP_000446.1:p.G163R	chr19:g.1220394G>C	VCV000182898
P179R	NP_000446.1:p.P179R	chr19:g.1220443C>G	—
S193Y	NP_000446.1:p.S193Y	chr19:g.1220485C>A	—
D194Y	NP_000446.1:p.D194Y	chr19:g.1220487G>T	VCV000007450
H202R	NP_000446.1:p.H202R	chr19:g.1220587A>G	VCV000480714
R211Q	NP_000446.1:p.R211Q	chr19:g.1220614G>A	VCV000182910
S216F	NP_000446.1:p.S216F	chr19:g.1220629C>T	VCV000376708
P221R	NP_000446.1:p.P221R	chr19:g.1220644C>G	—
A241P	NP_000446.1:p.A241P	chr19:g.1220703G>C	—
G242V	NP_000446.1:p.G242V	chr19:g.1220707G>T	—
G251C	NP_000446.1:p.G251C	chr19:g.1221228G>T	—
P275L	NP_000446.1:p.P275L	chr19:g.1221301C>T	VCV000480721
P280A	NP_000446.1:p.P280A	chr19:g.1221315C>G	VCV000458066
R297M	NP_000446.1:p.R297M	chr19:g.1221975G>T	—
R297S	NP_000446.1:p.R297S	chr19:g.1221976G>T	VCV000182912
W308R	NP_000446.1:p.W308R	chr19:g.1222985T>C	VCV000843663
K311N	NP_000446.1:p.K311N	chr19:g.1222996G>T	—
F354L	NP_000446.1:p.F354L	chr19:g.1223125C>G	VCV000007461
A397S	NP_000446.1:p.A397S	chr19:g.1226533G>T	VCV000127699
R409W	NP_000446.1:p.R409W	chr19:g.1226569C>T	VCV000135917
A417S	NP_000446.1:p.A417S	chr19:g.1226593G>T	VCV000142993

Nomenclature references human genome build GRCh37/hg19.

## Materials and methods

### Materials

Dulbecco’s modified eagle’s medium and RPMI 1640 were purchased from Thermo Scientific (Waltham, MA), fetal bovine serum was obtained from R&D Systems (Minneapolis, MN). Wild-type (WT; Addgene #8590) and kinase-dead (Addgene #8591) STK11 plasmids along with the p53-driven firefly luciferase (PG13-luc) and constitutive *Renilla* luciferase (pRL-SV40) plasmids, and the Cas9 and sgRNA plasmid used for asymmetric repair (pSpCas9(BB)-2A-GFP (PX458)) were obtained from Addgene (Cambridge, MA). Antibodies directed against LKB1 (E-9; #sc-374334), p53 (D0-1; #sc-126), Actin (C-2; #sc-8432), STRADα (G-8; sc 515635), MO25 (#2716) and Tubulin (#2144) were obtained from Santa Cruz Biotechnology (Dallas, TX) and Cell Signaling Technology (Danvers, MA), respectively. Secondary antibodies (α-mouse-horseradish peroxidase [HRP] and α-rabbit-HRP) were purchased from Jackson ImmunoResearch Laboratories (West Grove, PA). All other reagents were purchased from Fisher Scientific (Waltham, MA) unless otherwise noted.

### Authentication of cell lines

HEK 293, A549 and NCI-H441 cell lines were purchased directly from American Type Culture Collection (ATCC, Manassas, VA) and authenticated by short tandem repeat analysis in June 2019. Cells were expanded, cryopreserved at −190°C and only early passage cells (<passage 6) were used for this work. A549 lung adenocarcinoma cells harbor a nonsense mutation pQ37* and are STK11 null.

### STK11 variant identification

STK11 splice-site and missense variants were identified in collaboration with the Genomic Medicine Service at the University of Vermont Medical Center. In total, four STK11 splice-site variants and 28 STK11 missense variants (Table 1) were selected for characterization.

### Evaluation of STK11 splice-site variants in primary tumors by RT-PCR amplicon sequencing

**Figure 1.**
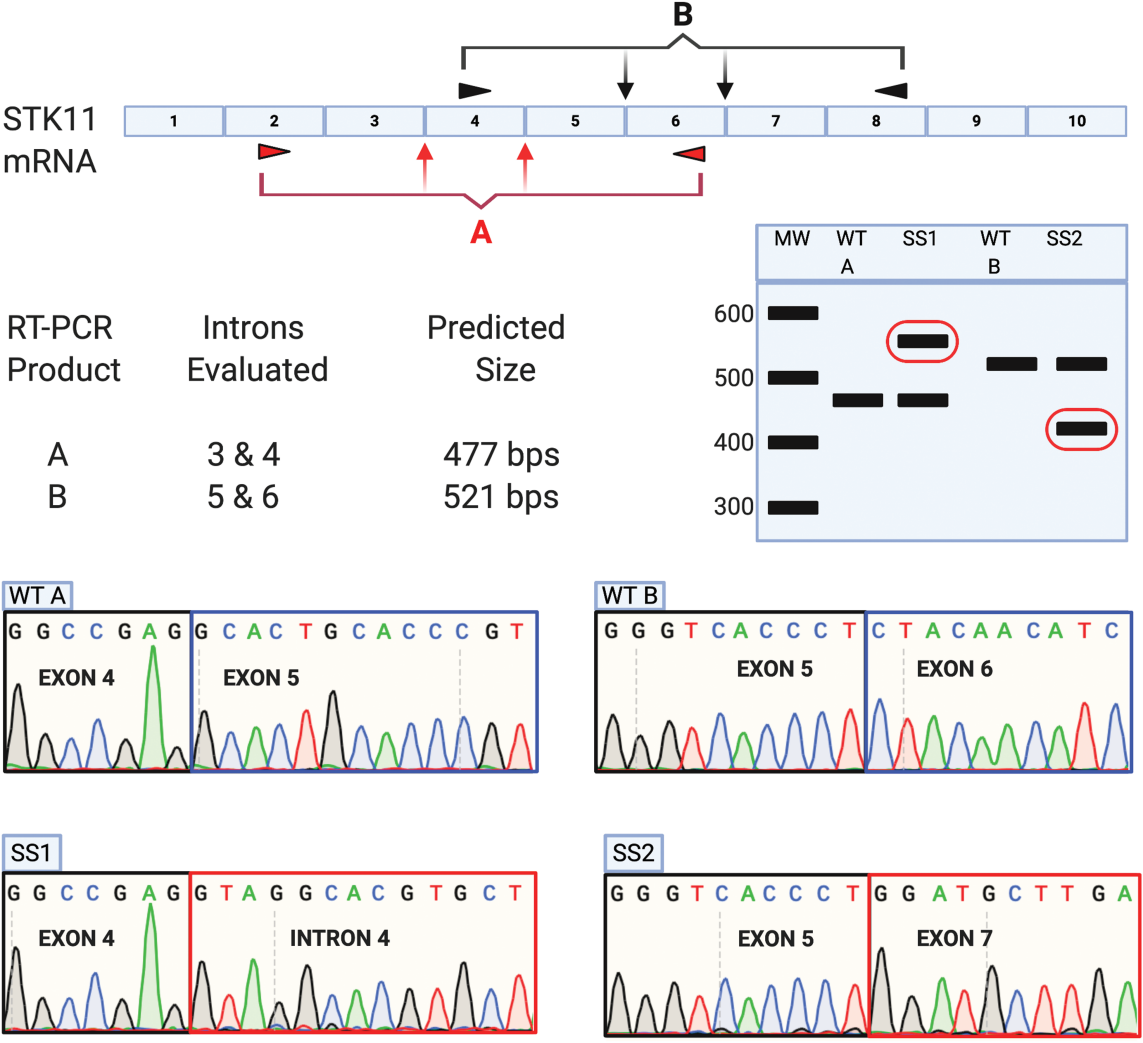
Approach utilized to assess STK11 mRNA isolated from NSCLCs harboring STK11 splice-site variants using RT-PCR followed by Sanger Sequencing. Nucleic acids harvested from NSCLC samples harboring STK11 splice-site variants were utilized as template in RT-PCR reactions to determine the impact on STK11 mRNA splicing. Tumors with variants located in either introns 3 or 4 were analyzed using primer pair ‘A’, while those with variants in introns 5 or 6 were assessed with primer pair ‘B’. Reaction products were separated by gel electrophoresis. Products of unexpected size were excised and sequenced to determine the impact on STK11 mRNA production.

Total nucleic acid was extracted from pathologist-qualified tissue samples using the QIAamp DNA FFPE Tissue Kit (Qiagen, Inc.) and further separated into RNA and DNA components per manufacturer’s recommendations. Targeted reverse transcription–polymerase chain reaction (RT-PCR) was performed on the purified RNA using the NEB OneTaq One-Step RT-PCR kit (#E5315S) per manufacturer’s recommendation with primers designed to flank non-involved introns on both sides of the detected variant location. Primer sequences for each reaction are included in Supplementary Table 1 (available at *Carcinogenesis* Online) and assay design described in Figure 1. Reaction products were resolved by gel electrophoresis, excised and purified using the QiaQuick Gel Extraction Kit (#28704; Qiagen, Inc.) per manufacturer’s protocol and sequenced in the Vermont Integrated Genomics Resource Core facility at the University of Vermont by standard Sanger methods using the forward primer from the RT-PCR reaction.

### CRISPR-Cas9 homology-directed base editing

NCI-H441 cells (ATCC #HTB-174) were maintained in RPMI 1640 containing 10% fetal bovine serum and 2mM L-glutamine in a humidified environment at 37°C and 5% CO_2_. The STK11 c.465-2A>C (NM_000455.4 c.465-2A>C) splice-site variant was successfully reconstituted in the NCI-H441 cell line using CRISPR-Cas9 homology-directed base editing with an asymmetric donor template (1 nmole) co-transfected with 2.5 ug of the pSpCas9(BB)-2A-GFP (PX458) vector expressing Cas9, an sgRNA and GFP (Addgene #48138) using Lipofectamine 3000 (Invitrogen, Carlsbad, CA) following the manufacturer’s protocol. The sgRNA and repair template sequences were purchased from IDT (Integrated DNA Technologies, Coralville, IA) and are listed in Supplementary Table 1, available at *Carcinogenesis* Online. Transfected cells were clonally selected based on GFP expression using FLOW cytometry in the University of Vermont FLOW core. Once expanded, clones were screened for correct repair by targeted genomic PCR with locus specific primers (Ex4/5 GCD, Supplementary Table 1, available at *Carcinogenesis* Online) followed by Sanger sequencing using the Ex4/5 GCD forward primer.

### qRT-PCR to evaluate cell line STK11 mRNA expression

Total RNA was isolated from H441 cells using the thermo scientific GeneJET RNA Purificaiton Kit (cat# K0731) and following the manufacturer’s recommended protocol. STK11 mRNA levels were quantified on a QuantStudio 3 Real-Time PCR System (Applied Biosystems cat# A28136) using the sybr-based Luna Universal one-step RT-qPCR kit (New England Biolabs cat# E3005, Ipswich, MA) with two sets of STK11 exon-junction spanning primer pairs (Supplementary Table 1, available at *Carcinogenesis* Online). STK11 mRNA levels were normalized to PSMB4 expression (33).

### Site directed mutagenesis

Variants of unknown significance were reconstituted using pcDNA3-FLAG-WT and the Q5 Site Directed Mutagenesis Kit (New England Biolabs, Ipswich, MA) following manufacturer’s protocol. Briefly, DNA primers containing a single nucleotide polymorphism or flanking a deleted segment were designed using NEBcloner.com. PCR was utilized to incorporate the change into a vector encoding WT-STK11. The PCR product was ligated and transformed into *Escherichia coli* (DH5α). Variant STK11 alleles were confirmed via Sanger sequencing performed at the Vermont Integrative Genomics Resource at the University of Vermont. Primers used to generate each variant are listed in Supplementary Table 2, available at *Carcinogenesis* Online.

### Kinase activity assay: STK11 autophosphorylation activity measured by gel-shift assay

Plasmids (described above) containing cDNAs encoding each of the mutant STK11 alleles fused to a FLAG-tag were co-transfected into HEK293 cells along with STRADα. STK11 heterotrimeric complexes (STK11/STRADα/MO25) were IP’d using anti-Flag beads and Kinase assays performed (± adenosine triphosphate [ATP], ± lambda phosphatase). Briefly, immunoprecipitates were washed three times with NP40, and twice with kinase wash buffer (50 mM Tris, pH 7.5, 10 mM MgCl_2_). After final wash, immunprecipitates were resuspended in kinase reaction buffer (50 mM Tris, pH 7.5, 10 mM MgCl_2_, 1 mM DTT, 100 μM ATP, 50 mM Beta glycerol phosphate). ATP was omitted from kinase reaction buffer for the (−) ATP conditions. Samples were placed in a shaking incubater at 37°C for 90 min at 1000 rpm. Laemmli buffer was added to stop the reaction and samples were subjected to SDS-PAGE analysis using 4–20% gradient polyacrylamide gels (Bio-Rad Laboratories, Hercules, CA). Proteins were transferred to nitrocellulose and western blot analysis was performed using anti-STK11 antibody (E-9; #sc-374334, Santa Cruz Biotechnology; Dallas, TX) and visualized with anti-mouse-HRP purchased from Jackson ImmunoResearch Laboratories (West Grove, PA). Membranes were stripped and western blot analysis was performed using anti-STRADα (G-8, #sc515365, Santa Cruz Biotechnology; Dallas, TX) and anti-MO25 (C49D8; #2716, Cell Signaling Technology, Danvers, MS).

### p53-mediated Luciferase Assay

A549 cells (ATCC #CCL-185) were maintained in Dulbecco’s modified eagle’s medium containing 10% fetal bovine serum and 2mM L-glutamine in a humidified environment at 37°C and 5% CO_2_. Five hundred nanogram of STK11/eGFP, 500 ng PG13-luc and 250 ng pRL-SV40 plasmid DNA (Addgene, Cambridge, MA) were added to cells with Lipofectamine 3000 (Invitrogen, Carlsbad, CA) following manufacturer’s protocol. Eighteen hours post transfection, cells were washed in 1× PBS and harvested using Passive Lysis Buffer provided with the Luciferase Assay Kit (Promega, Madison, WI). The luciferase assay was performed per manufacturer’s protocol and read using a SpectraMax M4 Plate Reader (Molecular Devices, San Jose, CA).

### Western blotting

Protein extracts were subject to SDS-PAGE electrophoresis and transferred to a nitrocellulose membrane using a Trans-Blot Turbo Transfer System (Bio-Rad Laboratories, Hercules, CA). Membranes were blocked for 1 h at room temperature in 1% bovine serum albumin in 1X Tris-buffered saline + 0.1% Tween-20 (TBST). Primary antibodies (LKB1 (E-9; #sc-374334), p53 (D0-1; #sc-126), Actin (C-2; #sc-8432), STRADα (G-8; sc 515635), MO25 (#2716) and Tubulin (#2144) were diluted 1:1000 in 1% bovine serum albumin in TBST and incubated overnight at 4°C. Membranes were washed three times in TBST for 10 min each prior to incubation with secondary antibody. Secondary antibodies were diluted 1:5000 (a-mouse-HRP) or 1:10 000 (a-rabbit-HRP) in TBST and rocked with the membrane for 1 h at room temperature. Membranes were subject to SuperSignal West Pico Chemiluminescent Substrate (Thermo Scientific, Waltham, MA) following three washes in TBST and imaged with a PXi4 EX imaging system (Syngene, Frederick, MD).

### *In silico* variant predictions

STK11 variant impact predictions were performed with 22 *in silico* algorithms (DANN, MutationTaster, Mutation Assessor, FATHMM, FATHMM-MKL, FATHMM-XF, LRT, DEOGEN2, EIGEN, EIGEN PC, SIFT, SIFT4G, PROVEAN, MVP, MutPred, REVEL, PrimateAI, MetaSVM, MetaLR, BayesDel addAF, BayesDel noAF, LIST-S2) using VarSome: The Human Genomic Variant Search Engine (34).

## Results

### STK11 splice-site variant assessment

Splice-site variants are often assumed to disrupt mRNA production, but there are many reports demonstrating this is not necessarily true (29–32). Despite recent improvements to *in silico* splice-site predictive tools (35,36), reliably assessing the functional impact of splice-site variants still requires direct assessment of mRNA. We designed an approach to evaluate *STK11* mRNA in tumors harboring clinically identified *STK11* splice-site variants. We utilized two separate RT-PCR reactions with primers targeting flanking exons: primer pair ‘A’ allows assessment of STK11 splice-site variants occurring in introns 3 and 4, while primer pair ‘B’ allows assessment of splice-site variants in introns 5 and 6 (Figure 1). If splicing of *STK11* is unaffected by a variant of interest then the predicted RT-PCR products for each reaction should be observed: 477 bps for product A, 521 bps for product B. If the variant disrupts *STK11* splicing, then the resulting RT-PCR product will be either larger or smaller than the predicted product (Figure 1, bands circled in red). It is important to note that the clinical tumor samples used for this analysis include non-tumor tissue. As a result, some level of the correct *STK11* RT-PCR products (A at 477 bps, or B at 521 bps) are expected.

**Figure 2.**
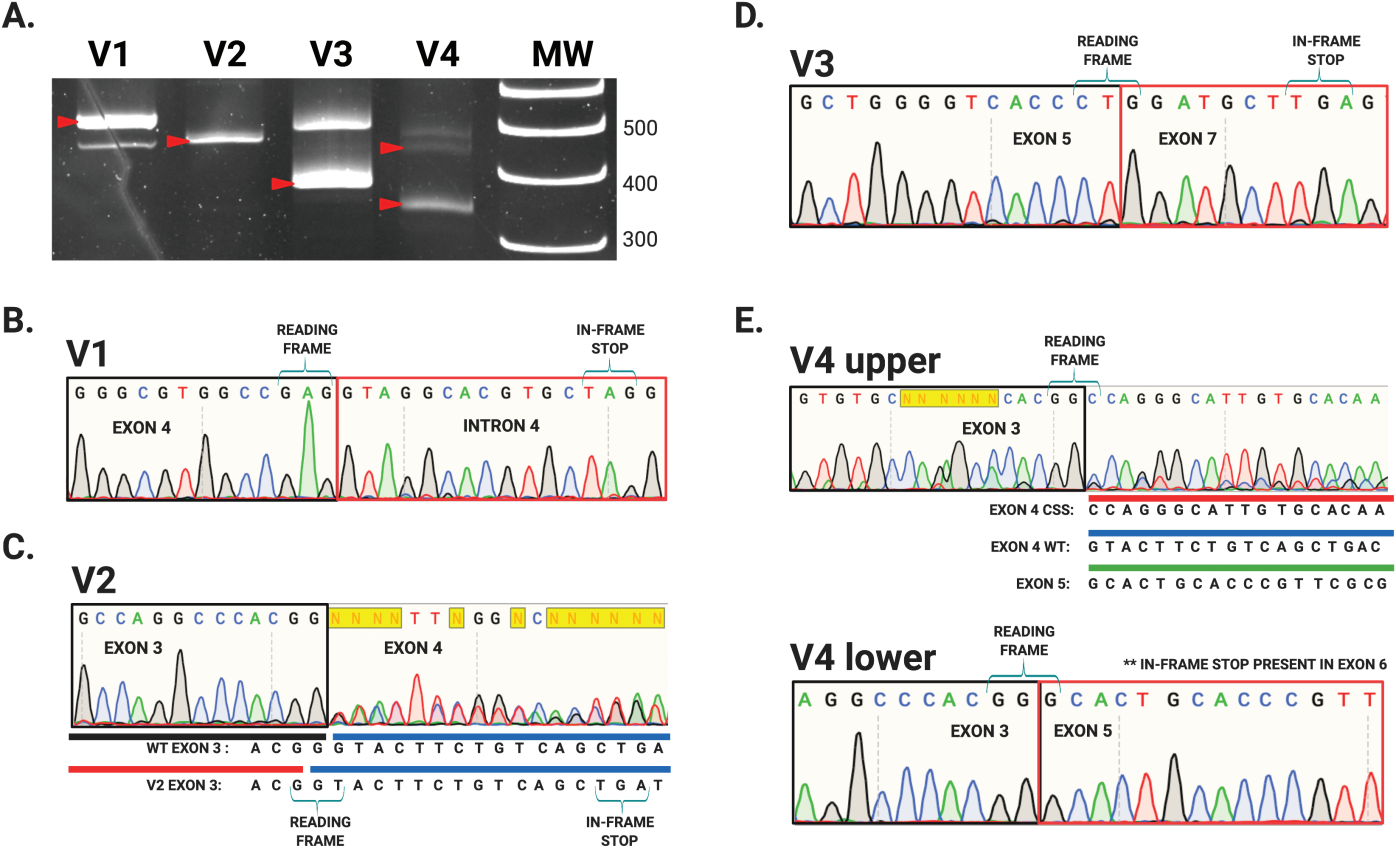
RT-PCR and Sanger sequencing reveals impact of STK11 splice-site variants from four human NSCLC samples. (**A**) Polyacrylamide electrophoresis demonstrates genotype-specific STK11 RT-PCR amplicons generated from different clinical samples. (**B**) Sample ‘V1’ (NM_000455.4 c.598-1G>A) produced an amplicon larger than WT (A, red arrow lane ‘V1’) resulting from intron 4 read-through. (**C**) Sample ‘V2’ (NM_000455.4 c.464+1G>T) generated an amplicon of predicted size (A, red arrow lane ‘V2’) which upon sequencing was revealed to represent an altered 5′ donor splice-site in intron 3. Interestingly, the G>T transition generates a new splice-site immediately upstream using the last ‘G’ of exon 3. The RNA effectively loses a G at this position, resulting in a frameshift and truncation. (**D**) Sample ‘V3’ (NM_000455.4 c.862+1G>A) produced a smaller than predicted amplicon (A, red arrow lane ‘V3’) demonstrated to result from exon 6 skipping. (**E**) Sample ‘V4’ produced 2 unexpected amplicons: an in-frame 42 bps deletion secondary to use of a cryptic splice-site (CSS) (A, top red arrow in lane ‘V4’ and E) as well as an amplicon resulting from exon 4 skipping (A, lower red arrow in ‘V4’). Exon 4 skipping is predicted to result in a frameshift, but the in-frame 42 bps deletion predicts a 14 AA in-frame deletion.

**Figure 3.**
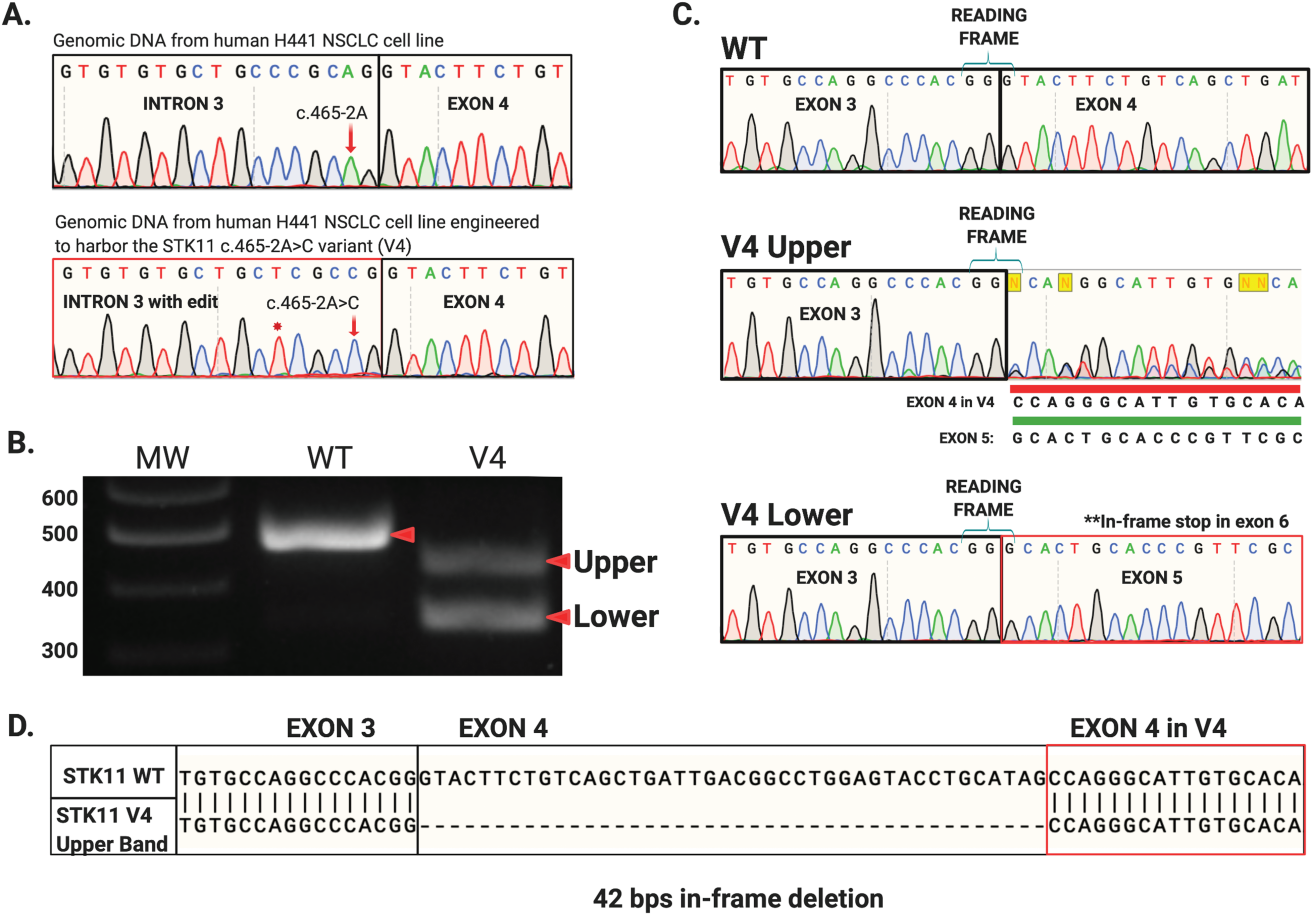
CRISPR-Cas9 homology-directed base editing using an asymmetric donor DNA template to reconstitute the V4 splice-site variant (NM_000455.4 c.465-2A>C) within the endogenous STK11 locus of NCI-H441 cells. (**A**) Following clonal selection by FLOW cytometry clones harboring the engineered c.465-2A>C allele were identified by targeted genomic sequencing. (**B**) RT-PCR performed with primer pair ‘A’ on mRNA isolated from these engineered cells produced STK11 products identical to those documented in the corresponding clinical V4 sample, labeled ‘upper’ and ‘lower’. (**C**) Sequencing these amplicons revealed they matched the mRNA species identified in the V4 clinical sample (Figure 2E). (**D**) The “upper” band represents utilization of a cryptic splice acceptor site within STK11 exon 4 generating an in-frame STK11 mRNA with a 42 bps deletion.

We performed our assay on mRNA isolated from primary tumors harboring each of the four identified *STK11* splice-site variants (Table 1). Detectable RT-PCR products either larger or smaller than the predicted sizes were observed for variants V1, V3 and V4 (Figure 2A, red arrows). Two of the variants, V1 and V4, occurred at STK11 splice acceptor sites: V1 in intron 4, and V4 in intron 3. Variant V2 and V3 occurred at splice donor sites in introns 3 and 6 respectively (Table 1). Interestingly, variant V2 produced a single uniform product of predicted size for primer pair A, suggesting the alteration did not impact *STK11* splicing (Figure 2A, lane ‘V2’ red arrow). To define the impact of these splice-site variants, we purified the resulting RT-PCR amplicons and sequenced them (Figure 2). Variant V1 resulted in a larger than expected product (>477 bps, Figure 2A, lane V1 red arrow) and sequencing revealed this variant (NM_000455.4 c.598-1G>A) caused intron 4 read-through. Inclusion of intron 4 in the *STK11* mRNA generates an in-frame stop codon that truncates the resulting protein (Figure 2B). The single band produced by variant V2 (NM_000455 c.464+1G>T) ran at the predicted size and therefore we *initially* expected no impact on the *STK11* mRNA sequence (Figure 2A, lane V2 red arrow). However, sequencing revealed the G > T transversion effectively shortened *STK11* exon 3 by one base pair via generation of a new splice donor sequence adjacent to the original. As a result, even though exon 3 is efficiently spliced to exon 4, the elimination of a base causes a frameshift and subsequent stop early in exon 4 (Figure 2C). Variant V3 (NM_000455.4 c.862+1G>A) generated a smaller than expected product (<521 bps, Figure 2A, lane V3 red arrow). Sequencing revealed this band represented exon 6 skipping which leads to a frameshift and in-frame stop codon (Figure 2D). Finally, variant V4 (NM_000455.4 c.465-2A>C) generated two unexpected RT-PCR amplicons, both smaller than the WT *STK11* mRNA (Figure 2A, lane V4 red arrows). The V4 ‘lower’ band was sequenced and found to represent exon 4 skipping, which creates a frameshift and stop codon in exon 6 (Figure 2E). The ‘upper’ V4 band was determined to arise from utilization of a cryptic splice acceptor site within exon 4. Intriguingly, this cryptic splice-site retained the correct reading frame for the *STK11* transcript. The result was an *STK11* mRNA with an in-frame 42 bps deletion (Figures 2E and 3D). In theory this mRNA should produce an STK11 protein with an in-frame 14 amino acid deletion. Unfortunately, we could not assess protein levels in the corresponding clinical sample. To address this limitation we employed CRISPR-Cas9 homology-directed base editing using an asymmetric donor template (37) to engineer the V4 variant into the endogenous *STK11* locus of NCI-H441 cells, thus allowing us to investigate the impact of the V4 variant in greater detail.

**Figure 4.**
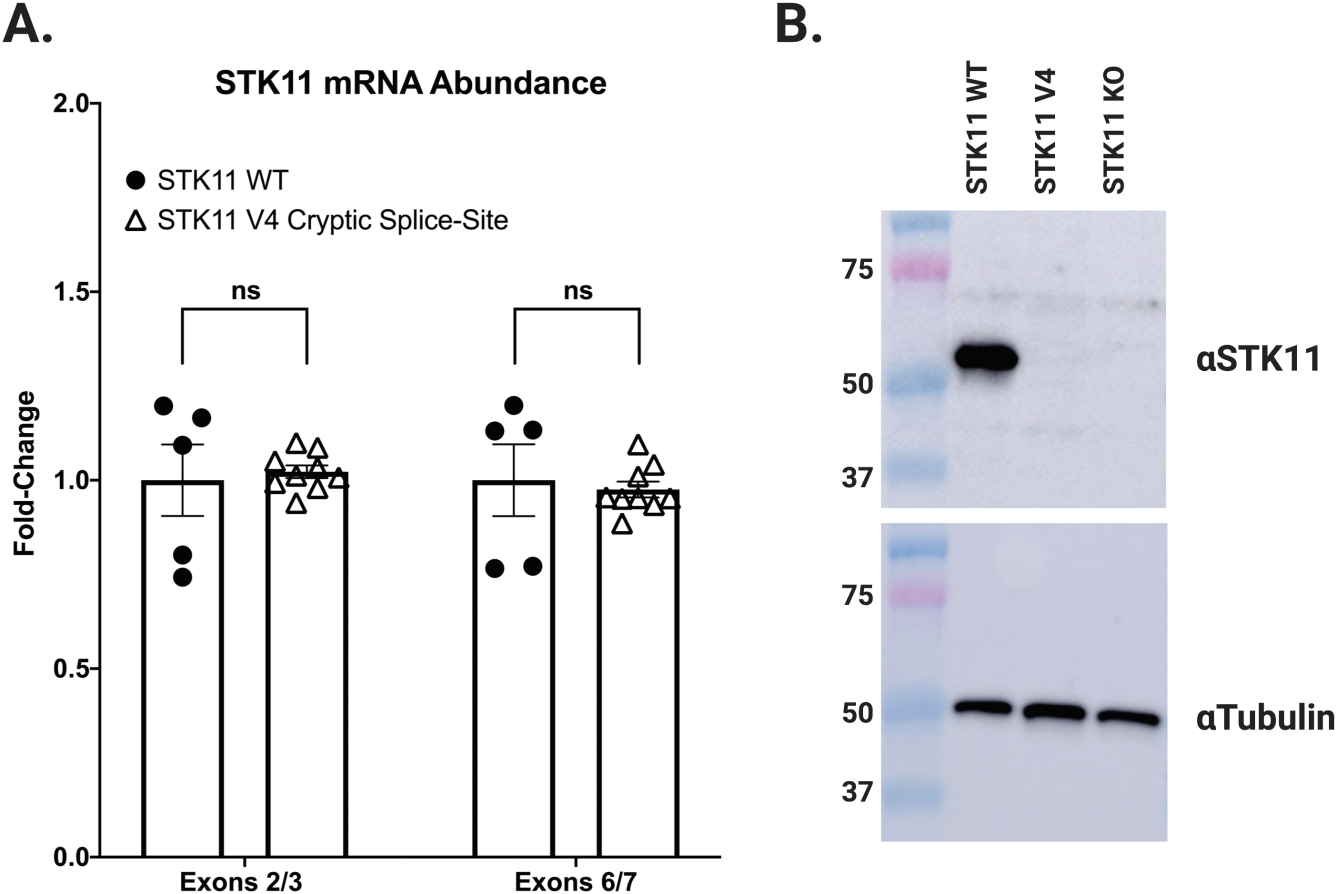
*STK11* mRNA arising from utilization of the exon 4 in-frame cryptic splice-site (V4) does not produce a detectable STK11 protein. (**A**) NCI-H441 cells edited to express the *STK11* c.465-2A>C variant (V4) produce equivalent amounts of *STK11* mRNA (open triangles) compared with NCI-H441 WT cells (closed circles). (**B**) Despite expressing equivalent amounts of *STK11* mRNA, no STK11 protein is detected in cells expressing the *STK11* c.465-2A>C variant by western blot using an antibody specific to the STK11 N-terminus.

NCI-H441 cells are derived from a human NSCLC with WT *STK11* alleles. As shown in Figure 3A, we successfully generated a clonal cell line where both *STK11* alleles harbored the V4 variant (NM_000455.4 c.465-2A>C; Figure 3A, red arrow). When *STK11* mRNA from this cell line was interrogated by RT-PCR with primer pair A, no WT band was detected, only bands corresponding to V4 ‘upper’ and ‘lower’ bands (Figure 3B). Sequencing these bands revealed they matched perfectly with the amplicons identified from the corresponding tumor harboring the V4 variant; the ‘lower’ band representing exon 4 skipping, and the ‘upper’ band resulting from exon 4 cryptic slice-site utilization (Figures 2E and 3C). We then compared the relative abundance of WT *STK11* mRNA in the parent H441 cell line to the base-edited cell line engineered with both *STK11* alleles expressing the V4 variant. This was accomplished using quantitative RT-PCR with primer pairs targeting STK11 exons 2/3 and 6/7 (Supplementary Table 1, available at *Carcinogenesis* Online). Analysis revealed no significant difference in *STK11* mRNA abundance between the parent and V4 variant cell lines (Figure 4A). This result indicates that the amount of *STK11* mRNA produced by the V4 variant locus was essentially equivalent to that produced by the WT locus. However, when protein was isolated from these two cell lines and subjected to western blot analysis with an antibody targeting the STK11 N-terminus, no STK11 protein was detected in the V4 variant expressing cell line (Figure 4B). This unexpected finding suggests the 14 AA deletion predicted to result from utilization of the *STK11* exon 4 cryptic splice-site likely renders the protein unstable. Ultimately, all four splice-site alterations we detected and evaluated resulted in disruption of *STK11* mRNA marked by either intron read-through (V1), exon skipping (V3) or cryptic splice-site utilization (V2 and V4). Further, our results demonstrate that predicting splicing outcomes for splice-site variants based solely on primary sequence data remains an unconquered challenge.

### Functional assessment of STK11 missense variants

**Figure 5.**
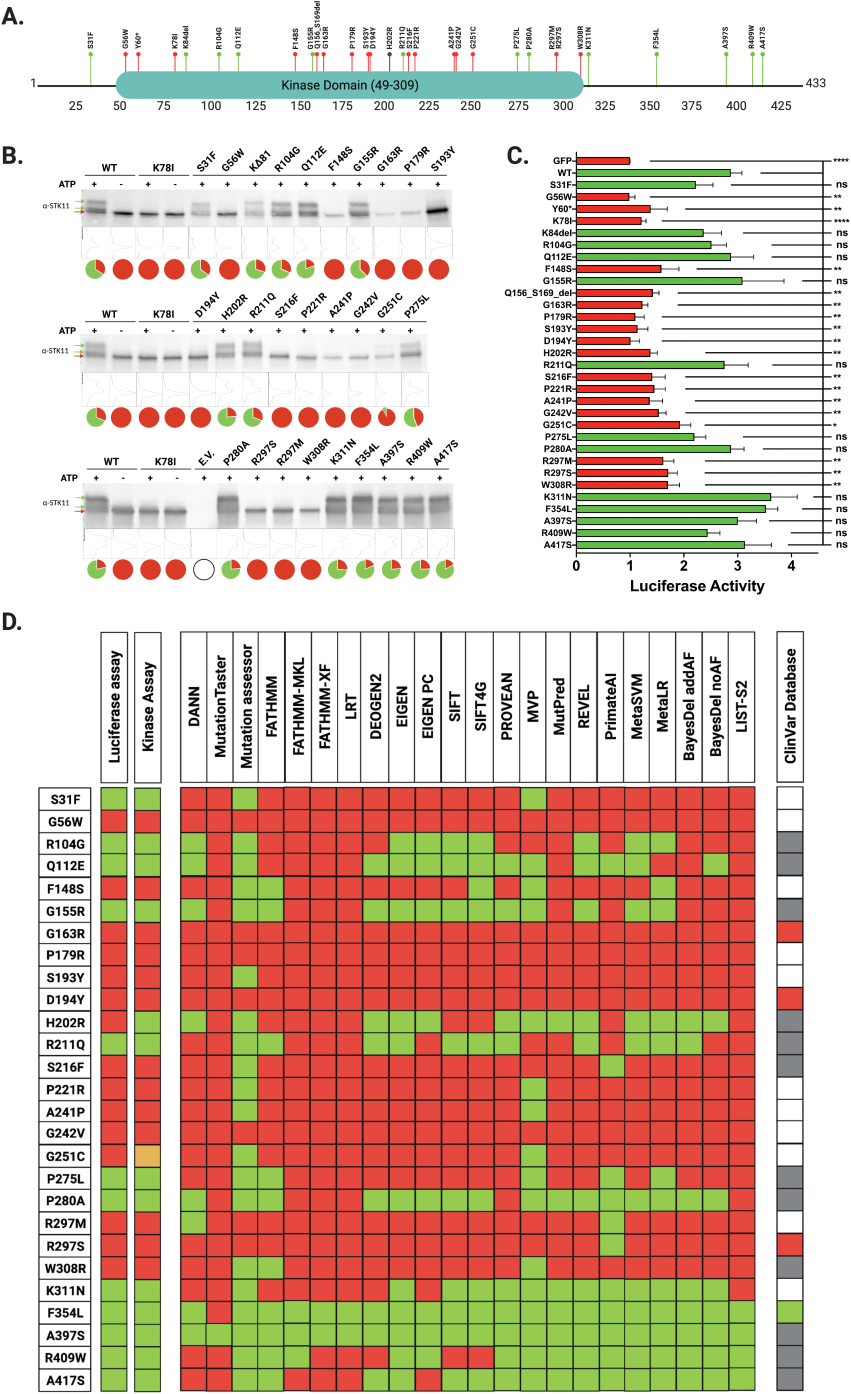
Location and functional impact of 28 clinically identified STK11 missense variants. STK11 missense variants represent the most common class of somatic variant and are also the most difficult to assess. We sought to evaluate the functional impact of clinically identified STK11 missense variants by using orthogonal approaches including an STK11 autophosphorylation assay and a luciferase-based p53 transcriptional activation assay. (**A**) The locations of each assessed variant relative to the STK11 polypeptide chain are represented by labeled bubbles where ‘red’ indicates significant loss of functional activity in both assays relative to WT STK11, while ‘green’ represents no significant change in activity relative to WT. ‘Gray’ represents conflicting results. (**B**) Protein from HEK293 cells transfected with Flag-tagged STK11 constructs was isolated and subjected to immunoprecipitation with anti-Flag beads. Purified protein complexes were then subjected to our *in vitro* kinase assay with (+) and without (−) ATP. Controls were treated separately with lambda phosphatase (Supplementary Figure 1, available at *Carcinogenesis* Online). All reactions were analyzed by SDS-PAGE and western blot with STK11 antibody. The electrophoretic mobility shift (green arrows) indicates STK11 autophosphorylation. The pie charts associated with each lane display the relative % phosphorylated STK11 (green) versus unphosphorylated STK11 (red) determined by ImageJ analysis. Lane plots from ImageJ are included between the blots and pie charts. (**C**) STK11-dependent p53-mediated luciferase activity is plotted relative to the GFP empty vector control. Each variant was analyzed with respect to STK11 WT signal by unpaired student t-test (*n* ≥ 5 for all samples, *P* ≤ 0.05). (**D**) We compared the luciferase-based functional assay and kinase assay results to 22 *in silico* predictive algorithms and the ClinVar database; ‘red’ boxes indicate a likely pathogenic prediction and “green” boxes indicate a likely benign prediction; for ClinVar, ‘gray’ boxes indicate conflicting reports or uncertain significance, while ‘white’ boxes indicate no information available. Only two of the variants resulted in uniform agreement across all predictive algorithms while also correlating with our functional results and the ClinVar database (G163R, D194Y). Four additional variants resulted in uniform agreement across all predictive algorithms while also correlating with our functional results, but were either absent from ClinVar, or reported as uncertain significance or conflicting impact (G56W, P179R, G242V and A397S).

Establishing the functional impact of novel missense variants continues to limit the implementation of comprehensive personalized genomic medicine. The reasons for this are varied, but generally reduce to the time-intensive efforts and expense necessary to generate reliable data. To address the need for rapid and dependable missense variant interpretation, numerous *in silico* predictive algorithms have been developed to evaluate the likelihood that a given alteration will impact protein function (38). Unfortunately, application of these tools reveals they rarely yield concordant results with respect to pathogenicity predictions, evidenced by the 28 *STK11* missense variants we evaluate herein (Table 1 and Figure 5D). To experimentally ascertain functional impact, we utilized complementary methods focusing first on STK11 kinase activity, and second on a well-characterized STK11-dependent signaling pathway, namely p53-mediated transcriptional activation.

### Kinase activity: STK11 autophosphorylation activity as measured by gel-shift assay

Flag-tagged plasmids encoding cDNAs for each of the STK11 missense variants (Table 1) were transfected into HEK293 cells. STK11 heterotrimeric complexes (STK11/STRADα/MO25) were then immunoprecipitated using anti-Flag beads and in vitro kinase assays performed. The reactions were subjected to SDS-PAGE and transferred to nitrocellulose membranes, followed by Western Blot analysis with an anti-STK11 antibody. Imaging the blots revealed two distinct banding patterns. In the positive control (Figure 5B, WT STK11), three ATP-dependent bands were observed, the lowest molecular weight (MW) band being unmodified STK11 (red arrow), while the two higher MW bands represent degrees of STK11 autophosphorylation (Figure 5B, green arrows). The second pattern is represented in the negative control reaction attained using a well-described catalytically dead STK11 missense variant, p.K78I (39), resulting in a single unmodified STK11 band (red arrow). Our interpretation of the banding pattern is supported by treatment with λ-phosphatase, which eliminated the higher MW bands in the positive control, demonstrating their creation is the result of phosphorylation (Supplementary Figure 1, available at *Carcinogenesis* Online). Although the possibility that STK11 is being phosphorylated by another kinase cannot be entirely excluded, we favor the detected modifications are due to STK11 autophosphorylation as no modified STK11 bands were detected when the kinase-dead K78I variant was tested. Each of the 28 missense variants (Table 1) were subsequently expressed, IP’d, and evaluated. Missense variants which failed to produce a band-shift were classified as loss of kinase function, while those generating band-shifts mirroring WT were classified as WT-like (Figure 5B and D). In all, 13/28 variants (S31F, R104G, Q112E, G155R, H202R, R221Q, P275L, P280A, K311N, F354L, A397S, R409W, A417S) were found to retain autophosphorylation activity under the conditions employed in our *in vitro* kinase assay while 14/28 variants (G56W, F148S. G163R, P179R, S193Y, D194Y, S216F, P221R, A241P, G242V,G251C, R297M, R297S, W308R) lacked autophosphorylation activity. One of the tested variants, G251C, resulted in the detection of a weak band-shift (G251C, Figure 5B), suggesting an incomplete loss of kinase activity. In general, however, our STK11 autophosphorylation assay efficiently discriminates variants impacting kinase function.

### STK11-dependent p53-mediated transcriptional activation

To provide an orthogonal read-out of STK11 function generated by our *in vitro* autophosphorylation activity assay we next took advantage of an STK11-dependent, p53-mediated transcriptional activation assay previously published to evaluate the pathogenicity of *STK11* germline mutations associated with Peutz-Jeghers Syndrome (27,40,41). Each of the 28 variant *STK11* cDNAs (Table 1) were expressed in *STK11* null A549 lung adenocarcinoma cells and expression verified by western blot (Supplementary Figure 2, available at *Carcinogenesis* Online). P53-mediated transcriptional activation was then measured using a dual luciferase reporter system (pp53-TA-luc & pRL-TK) co-transfected with the STK11 variants (Table 1). As shown in Figure 5C, when p53-dependent luciferase signal was measured following expression of each STK11 variant and compared to signal induced by expression of WT STK11, the variants fell into one of two categories: either significantly less luciferase activity compared with STK11 WT (red bars), or no significant difference in luciferase activity when compared to STK11 WT (green bars). Importantly, changes in luciferase signal were not due to differential p53 expression (Supplementary Figure 2, available at *Carcinogenesis* Online). We then compared our p53-dependent functional assessment of each *STK11* missense variant to results from our in vitro kinase assay (Figure 5B). The results of the p53-mediated transcriptional activation assay and the putative autophosphorylation assay agreed for 27/28 variants (Figure 5D). The only discrepancy occurred for the p.H202R variant. The luciferase assay classified p.H202R as LoF (Figure 5C), while the *in vitro* kinase assay demonstrated that STK11 autophosphorylation was maintained (Figure 5B).

In comparing the results of our functional assessments with 22 *in silico* predictive algorithms (Figure 5D), only 6/28 variants demonstrated uniform agreement across all platforms (G56W, G163R, P179R, D194Y, G242V and A397S). However, there were several variants wherein the majority of predictions agreed with our assay results (15/28: Q112E, F148S, G155R, S193Y, S216F, P221R, A241P, G251C, P280A, R297M, W308R, K311N, F354L, R409W, A417A). In contrast, three variants resulted in a near 50/50 split among the predictive algorithms (R104G, H202R and R211Q), while two of the variants were predicted to behave exactly opposite of what we found in our functional assessment (S31F and P275L). Finally, to complement our analyzes we queried the ClinVar database (https://www.ncbi.nlm.nih.gov/clinvar/) for information regarding the likely pathogenicity of the *STK11* missense variants evaluated. ClinVar is a repository for germline variants, and reports *STK11* germline variants in the context of Peutz-Jeghers Syndrome. If a patient presents clinically as concerning for Peutz-Jeghers Syndrome, and a germline missense variant is identified in *STK11*, then the variant may be flagged as likely pathogenic. As shown in Figure 5D, three of the somatic variants we assessed have been reported in germline studies and flagged as either pathogenic (R297S) or likely pathogenic (D194Y and G163R), while one of the variants we assessed has been flagged as likely benign (F354L). Our functional assessments for these four variants, based on our functional assays, are in agreement with the ClinVar categorizations, as are the majority of predictive algorithms (Figure 5B–D). However, most of the somatic *STK11* variants we report lack definitive categorization in ClinVar and instead are listed as being of ‘Uncertain Significance’ or as having ‘Conflicting Interpretations’ (12/28, Figure 5D). Critically, eleven of the 28 variants we interrogate are absent from the ClinVar database (Table 1 and Figure 5D), and of these, our functional assessments indicate 9 (G56W, F148S, P179R, S193Y, P221R, A241P, G242V, G251C and R297M) represent LoF variants (Figure 5B and C).

## Discussion

Next-generation sequencing assays are now commonly used to evaluate tumor mutations for the purpose of directing targeted therapy in clinical oncology. As a result, the number of variants identified in critical cancer genes has increased well beyond established ‘hot-spot’ alterations. Despite this increased accounting of novel or rare variants, there is frequently little published information regarding functional consequence, and these alterations are typically lumped as VUS. Unfortunately, the clinical application of variant identification relies on the ability to assign a functional consequence to each identified variant and without adequate experimental follow-up these variant databases offer little clinical utility.

Approximately 15 000 NSCLC patients per year will harbor concurring mutations in *STK11* and *KRAS*, predicting resistance to anti-PD-1 monoclonal antibody therapy. Therefore, our ability to confidently assign functional deficits to novel somatic *STK11* variants is paramount. Nonsense variants can generally be classified definitively as LoF, especially when they occur early in the *STK11* coding region. However, other somatic variant classes, including splice-site variants and missense variants, are more problematic. Ultimately, accurately predicting the functional impact of splice-site and missense variants is essential to implementing personalized genomic medicine and remains an unmet need.

*STK11* variants that occur at conserved non-coding splice-sites are often assumed to disrupt splicing and lead to non-productive mRNA molecules, effectively recapitulating STK11 LoF. However, this assumption is rarely backed by experimental evidence. To confidently determine the functional consequence of splice-site variants the mRNA must be analyzed as predictions based solely on DNA sequence are unreliable (29–32). According to publicly available tumor sequencing databases, ~1–3% of all somatic *STK11* single nucleotide variants in NSCLC occur at predicted splice-sites (42). Here we demonstrate the functional impact of *STK11* splice-site variants can be established by simply sequencing RT-PCR amplicons from tumor mRNA (Figure 2). Our work provides direct evidence that the four *STK11* splice-site variants assessed result in non-productive *STK11* mRNA and should therefore be categorized as LoF variants. These result also highlight the added utility of tumor mRNA assessment when attempting to accurately classify variant impact.

While *STK11* splice-site variants are an important category of alteration in NSCLC they are still relatively rare compared with *STK11* missense variants. Further, the functional assessment of splice-site variants is relatively straightforward as it can be achieved by directly interrogating tumor mRNA. In contrast, the functional assessment of missense variants poses a more complex set of challenges. Traditionally, *in vitro* assays with recombinant proteins have been used to assess variant impact. This assumes the activity of the target protein is understood well enough to assess function, and that its behavior *in vitro* recapitulates that *in vivo*. While there are inherent limitations to this approach, it does provide a direct avenue to identify variants that alter protein function. Of the 28 clinically identified somatic *STK11* missense variants we evaluate here, 13 met criteria for STK11 LoF based on *in vitro* kinase activity (Table 1 and Figure 5B). Using an orthogonal STK11-dependent p53 transcriptional activation assay, the same 13 variants also displayed loss of STK11 function (Figure 5C, red bars). Interestingly, a fourteenth variant, p.H202R, displayed LoF in the p53 assay (Figure 5C) but retained kinase activity (Figure 5B), which is consistent with studies that have identified amino acid residues 172–221 as important for substrate recognition (43). Several of the variants that displayed loss of both kinase activity and a defect in the p53 assay are located within the kinase domain regions known to be important for ATP binding and enzyme catalysis (19,43) including G56W, F148S, G163R, P179R, S193R, D194Y and S216F.

An important factor to consider when analyzing the impact of variants on STK11 kinase activity is whether STRADα and MO25 are efficiently bound, as the heterotrimeric STK11-STRADα-MO25 complex is required to achieve full activation of the enzyme (19). Our analyses reveal that any variant found to be defective in binding to STRADα and/or MO25 also displayed loss of kinase activity and defects in the p53 assay (Supplementary Figure 3, available at *Carcinogenesis* Online). Interestingly, 6/28 variants analyzed in our study exhibit defects in binding to both STRADα and MO25 (F148S, G163R, P179R, A241P, G242V, W308R) while two variants at position R297 (R297M and R297S) did not bind to STRADα but retained the ability to interact with MO25 (Supplementary Figure 3, available at *Carcinogenesis* Online). These results are consistent with previous structural studies that identified key amino acids in mediating STRADα and/or MO25 binding including G163, G242, R297 and W308 (18,19,44).

Critically, our work reveals discrepancies between functional assay data and the predictive *in silico* tools used to evaluate *STK11* variant pathogenicity (Figure 5D). For example, only six of the 28 variants we assess resulted in uniform agreement across the 22 predictive algorithms and our functional assays (G56W, G163R, P179R, D194Y, G242V and A397S). Of these, only one is definitively classified by ClinVar as ‘pathogenic’ (R297S) while two others are labeled as ‘likely pathogenic’ (D194Y and G163R). Twelve of the 28 variants we assess are reported in ClinVar, but insufficient data relegate them to ‘Uncertain Significance’ or ‘Conflicting Report’ status. Our functional data suggest that two of these twelve variants result in STK11 LoF (S216F and W308R), while nine are likely benign (R104G, Q112E, G155R, R211Q, P275A, A397S, R409W, A417S). Incredibly, 11 of the 28 variants we report are absent from the ClinVar database, and of these, we find nine exhibit STK11 LoF (G56W, F148S, P179R, S193Y, P221R, A241P, G242V, G251C, R297M) while only two retain activity (S31F and K311N).

An important caveat regarding our findings for somatic STK11 missense variants is that our functional assays predominantly evaluate STK11 kinase function. There exists a large body of work characterizing the kinase-independent functions of STK11 and how those activities impact cellular adhesion and motility (45,46). In particular, the C-terminal domain of STK11 (residues 309–433) appears to regulate cell polarity and directional migration independent of STK11 kinase activity (46,47). It is important to note that five of the VUS characterized as ‘functional’ in this study reside within this region of STK11 (K331N, F354L, A397S, R409W, A417S). Our work does not address whether these variants alter cellular polarity and migration phenotypes. In this regard, the F354L variant has been previously shown to display full kinase activity but a defect in cell polarity and directional migration (46,47). Whether mutations in the C-terminal domain contribute to lung cancer progression and/or are associated with poor patient outcomes remains to be established.

In summary, our work provides functional data on 32 STK11 VUS that can be used to inform clinical decision making for NSCLC patients. While the utility and accuracy of *in silico* predictive algorithms continues to improve, they remain far from infallible. As a result, using *in silico* assessment alone to triage large variant lists should be employed with caution. The need for rigorous and accurate characterization of STK11 functional status is now urgent as clinical decisions governing the use of anti-PD-1 monoclonal antibody therapy for patients with KRAS-driven lung adenocarcinoma have come to depend upon accurate STK11 functional evaluation. Additionally, for proteins with multiple cellular roles, such as STK11, functional and predictive assessments must expand to consider the impact of all variants that may help to guide patient prognosis and treatment. In the case of STK11, these must include those variants that lie outside the kinase domain, specifically those within the C-terminal domain. This area of research deserves additional focused attention, work that we and other groups continue to pursue. In the future, we anticipate that understanding the molecular interactions driving the clinical observation that STK11 loss correlates with resistance to anti-PD-1 therapy will reveal opportunities to restore sensitivity to immune therapy in these patients.

## Supplementary Material

bgab104_suppl_Supplementary_Figure_S1

bgab104_suppl_Supplementary_Figure_S2

bgab104_suppl_Supplementary_Figure_S3

bgab104_suppl_Supplementary_Table_S1

bgab104_suppl_Supplementary_Table_S2

## Abbreviations

ATP: adenosine triphosphate
LoF: loss of function
NSCLC: non-small cell lung cancer
RT-PCR: reverse transcription–polymerase chain reaction
STK11: Serine/Threonine Kinase 11
VUS: variants of uncertain significance
