# Supplementary figures and images for: Functional assessment of somatic STK11 variants identified in primary human non-small cell lung cancers
Source: Carcinogenesis. 2021 Nov 19;42(12):1428–38. doi: 10.1093/carcin/bgab104 (PMC8727739; doi:10.1093/carcin/bgab104)

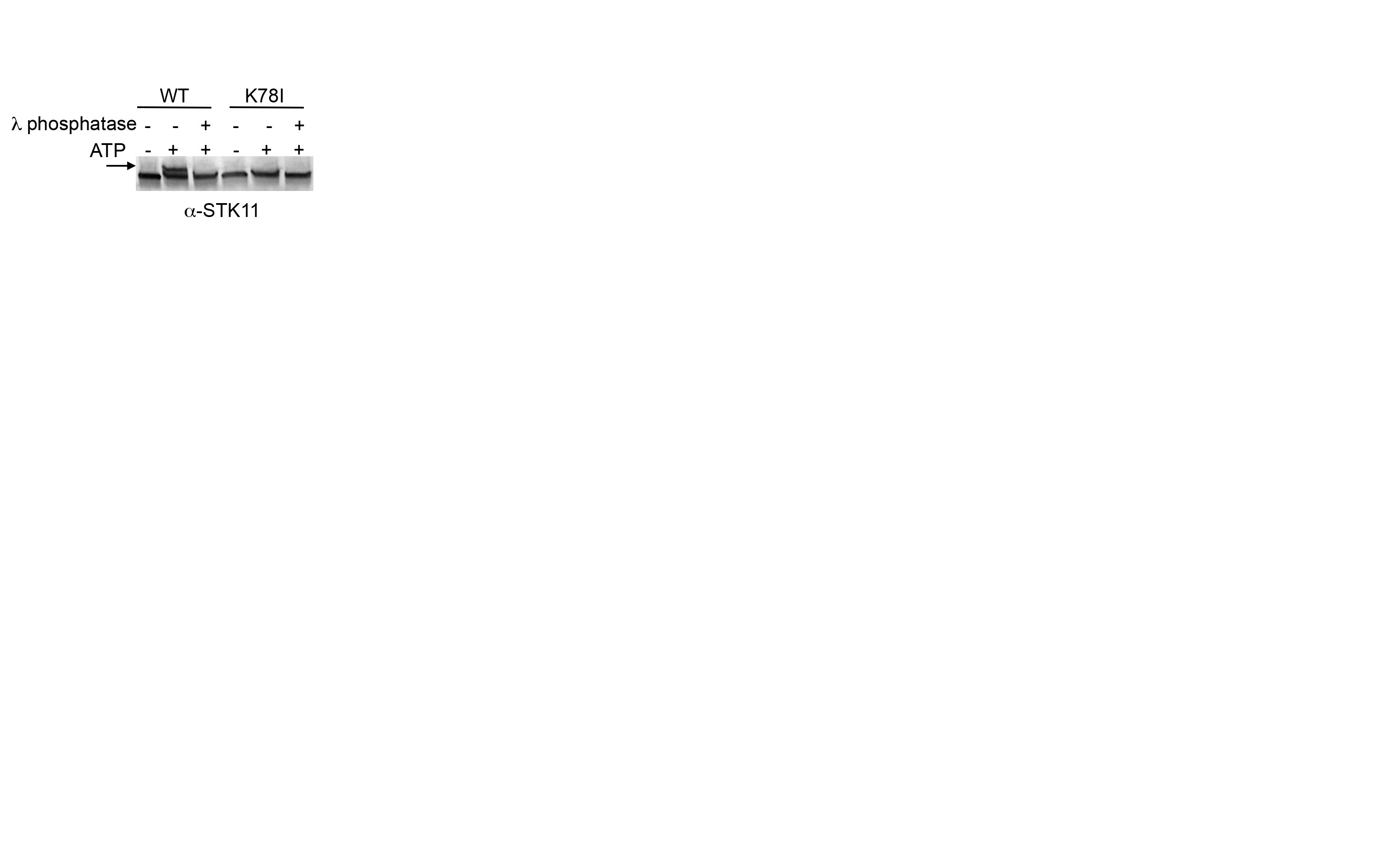

Supplement: bgab104_suppl_Supplementary_Figure_S1 [file bgab104_suppl_supplementary_figure_s1.jpeg]

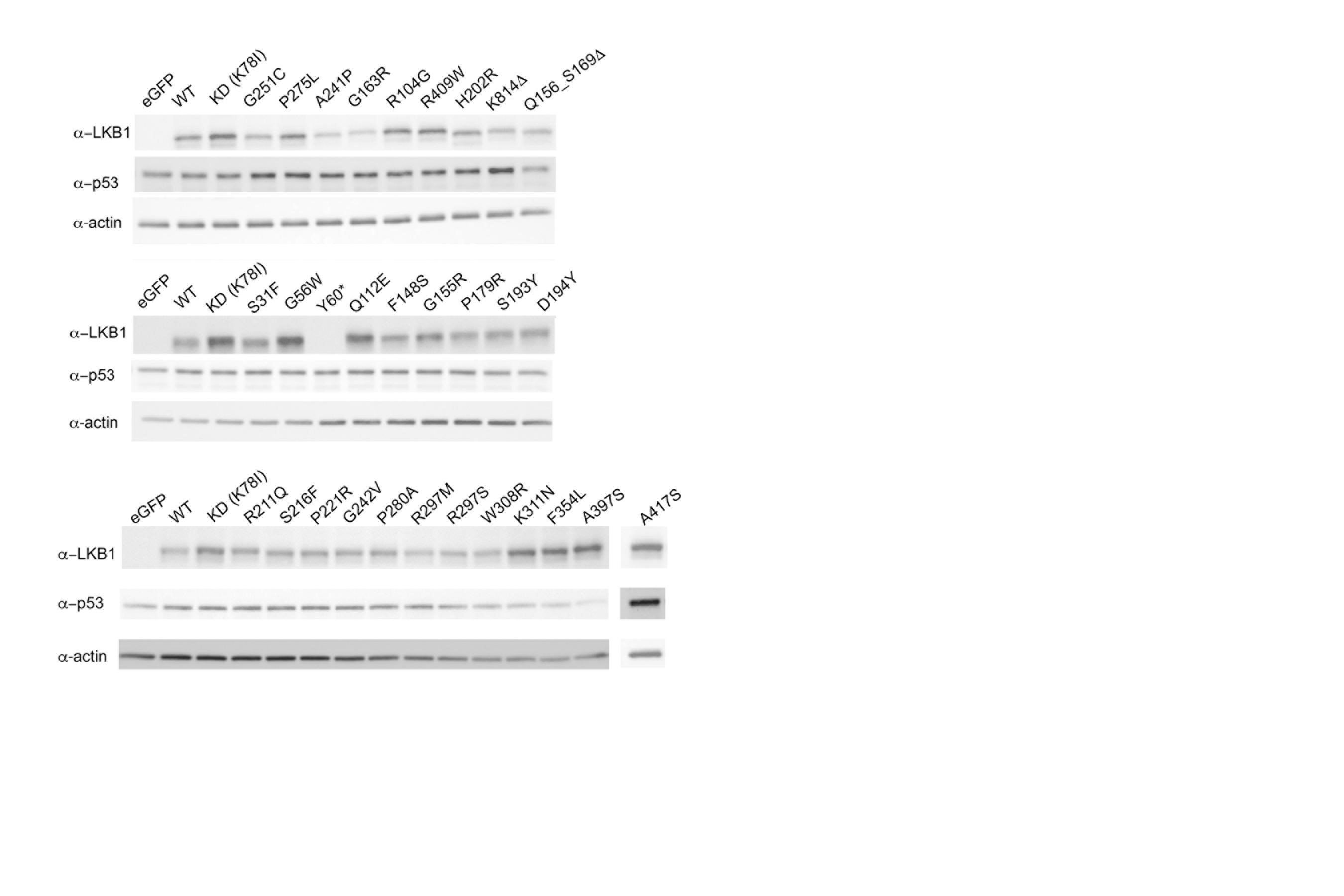

Supplement: bgab104_suppl_Supplementary_Figure_S2 [file bgab104_suppl_supplementary_figure_s2.jpeg]

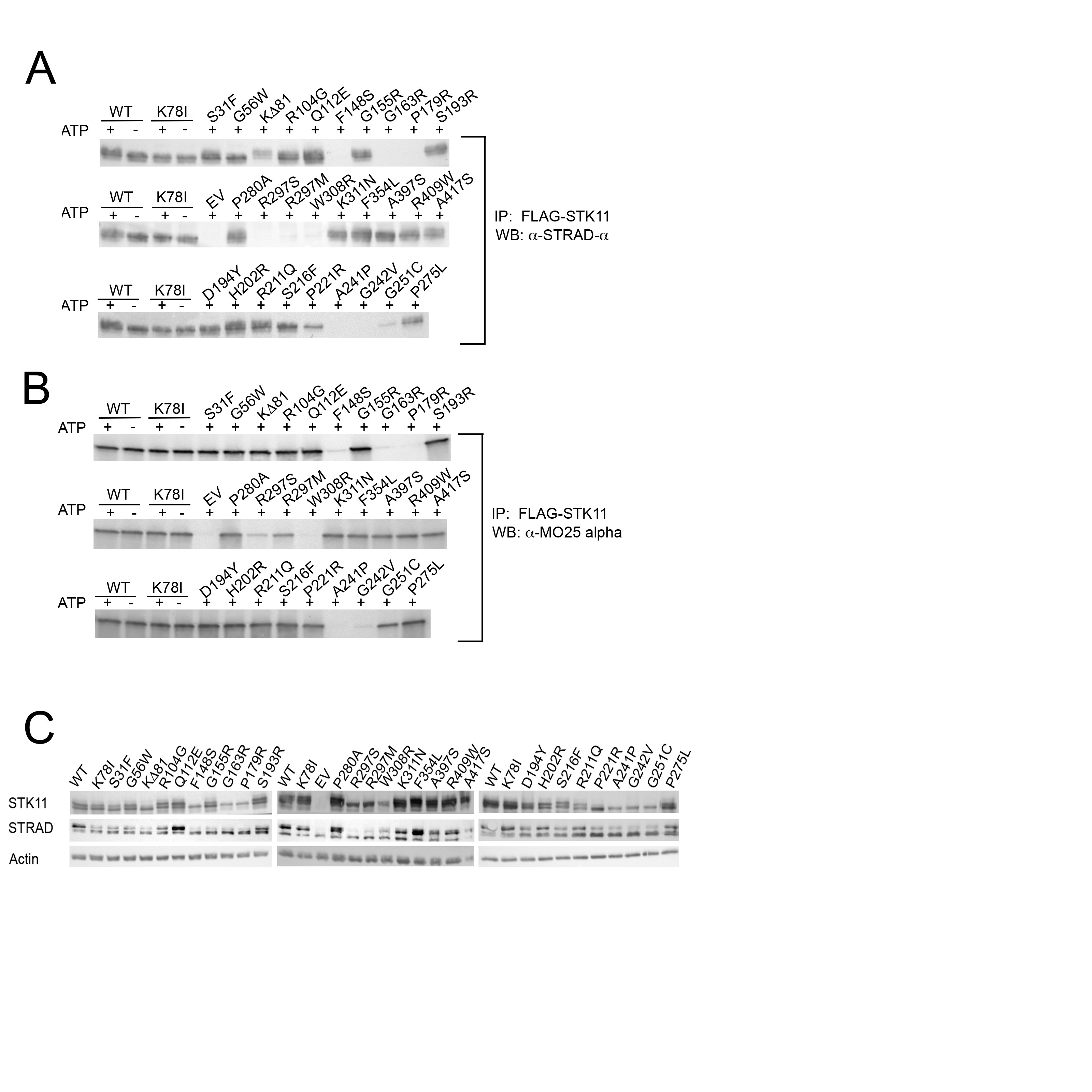

Supplement: bgab104_suppl_Supplementary_Figure_S3 [file bgab104_suppl_supplementary_figure_s3.jpeg]
